# Comparative study of the glucose and trehalose addition on the extenders for goat sperm liquid storage

**DOI:** 10.5455/javar.2025.l895

**Published:** 2025-03-26

**Authors:** Md. Emtiaj Alam, Most. Shorifa Yeasmin, Dipak Kumar Das, Md. Shohidul Islam, Soshe Ahmed, Md. Hamidul Islam, Md. Akhtarul Islam, Md. Mostofa Kamal, Aurangazeb Kabir, Alam Khan, Md. Hakimul Haque, Md. Abdul Masum, Moizur Rahman, Mst. Ishrat Zerin Moni

**Affiliations:** 1 Department of Veterinary and Animal Sciences, Faculty of Veterinary and Animal Sciences, Rajshahi University, Rajshahi, Bangladesh; 2 Department of Pharmacy, Faculty of Sciences and Engineering, East West University, Dhaka, Bangladesh; 3 Department of Pharmacy, University of Rajshahi, Rajshahi, Bangladesh; 4 Department of Anatomy, Histology and Physiology, Faculty of Animal Science and Veterinary Medicine, Sher-e-Bangla Agricultural University, Dhaka, Bangladesh

**Keywords:** Glucose, trehalose, diluents, semen, cold storage, spermatozoa

## Abstract

**Objectives:**

The objective of this study was to assess the effects of varying glucose and trehalose concentrations on tris-citric acid-egg yolk-fructose (TCEF) diluents for the short-term cold storage of goat semen.

**Materials and Methods:**

The semen sample was collected, unwashed, and divided into the following groups: control (TCEF without glucose and trehalose), TCEF + glucose (75, 150 mm), and TCEF + trehalose (75, 150 mm). Each experimental sperm group (sperm concentration: 9 × ×10^7^/ml) was kept at 4°C in a refrigerator. The impact of varying glucose and trehalose levels on the quality of the spermatozoa was assessed at different time points: after dilution and at 5, 24, 48, and 72 h of refrigeration.

**Results:**

After dilution, progressive motility (PM), total motility (TM), sperm viability (SV), functional integrity (FI), and acrosome integrity of G-75, G-150, T-75, and T-150 did not differ significantly from the control. The PM, TM, SV, FI, and acrosome integrity of sperm of T-150 were considerably lower than the control, G-75, G-150, and T-75 after 5 and 24 h of cool storage. The T-75 group showed superior PM, TM, and FI after 48 h of cool storage, with noticeably greater values than the other groups.

**Conclusion:**

This study indicates that trehalose is a more favorable sugar than glucose for 48 h cool storage of buck semen, providing greater advantages in PM, TM, and PMI.

## Introduction

Artificial insemination (AI) in goats is a reproductive technique that includes the collection of a buck’s sperm and its subsequent placement into the reproductive tract of a doe for fertilization. AI has been used in goat breeding programs to enhance genetic progress, improve breeding efficiency, and overcome logistical challenges associated with natural mating. Freezing sperm is an expensive operation, whereas liquid-stored sperm may be a better option for AI than cryopreserved sperm. However, as a result of osmotic stress, cold shock, and the generation of reactive oxygen species during the freezing procedures, sperm motility (SM), membrane integrity, and fertilizing capacity were reduced [1,2].

Among the various extenders utilized for the liquid storage of goat semen, the tris-citric acid-egg yolk-fructose (TCEF) diluent has emerged as a promising medium for short-term preservation due to its ability to maintain semen motility and viability. Whereas, optimizing the composition of extenders to enhance their efficacy remains an ongoing area of research. According to several studies, after being stored at a cool temperature, mammalian sperm exhibit improved motility, vitality, plasma membrane integrity, and acrosome integrity when sugar is added to an extender [3,4].

In addition, some studies have employed fructose or glucose in the tris-citric acid extender for mammalian semen freezing [5–9]. Fructose is a necessary component of glycolysis in buck seminal plasma; therefore, it is sensible to add it to the media [10]. Contrarily, glucose is a remarkable substrate for the metabolism of buck spermatozoa and is essential for the spermatozoa’s energy source in order for them to operate normally [9]. Fructose and glucose can flow through the plasma membrane of semen because of their low molecular weight.

Additionally, it has already been reported that monosaccharides (fructose and glucose) in rams have better freeze protection qualities than disaccharides (lactose, sucrose, or trehalose) [11]. In contrast to the simple sugars fructose and glucose, trehalose has been added to semen extenders. Trehalose stabilizes the lipid membrane, protein, and biofilm systems. It was renowned for its protective ability during the freezing of spermatozoa, preventing the growth of intracellular ice crystals, helping mitigate cellular osmotic stress-induced injury and temperature variations, maintaining protein structure, greatly enhancing sperm cell mobility, stabilizing the acrosome and cell membrane, and minimizing sperm cell abnormalities [12]. Trehalose has been demonstrated to have a key part in sperm preservation. Still, it was discovered that when trehalose content increased, the viscosity of the culture medium increased as well, which interfered with sperm mobility [13]. The specific concentration of each sugar that best preserves the quality of semen during liquid storage in the goat has not been currently established, and the individual effects of glucose and trehalose addition to the TCEF extender in goat semen cold storage preservation have not been extensively investigated.

The objective of this study was to optimize the formulation of TCEF diluents to improve their preservation efficacy by assessing the influence of varying concentrations of glucose and trehalose on the short-term chilled (4°C) storage of goat semen.

## Materials and Methods

### Ethical approval

The Rajshahi University Institutional Animal, Medical Ethics, Biosafety, and Biosecurity Committee (IAMEBBC) gave its approval for this study.

### Selection and management of animals

In this experiment, three adult beetal bucks were used. Their ages ranged from 28 to 40 months, weighing more than 50 kg. The University of Rajshahi, Veterinary and Animal Sciences Faculty runs the Naricalbaria, a center for veterinary clinics and an AI center, where the bucks are housed and provided care. In addition to receiving enough fresh water and midday sunlight, the experimental bucks were fed 600 gm of concentrate, 450 gm of paddy straw, and 2.5 kg of Napier grass every day.

### Collection of seminal samples

The criteria used for semen collection and selection were followed in accordance with Kamal et al. [14 and 15], with minor adjustments. In summary, the buck was stimulated by a doe, and an artificial vagina was used to obtain semen, ≥ 5 days apart. Semen was examined under a microscope after collection, and the selection parameters were determined. Ejaculates that met the following criteria were selected for this study: color creamy white, volume ≥ 0.65 ml, mass movement ≥ 3.20, concentration ≥ 2 × 10^9^ sperm/ml, SM ≥ 80%, sperm viability (SV) ≥ 80%, sperm plasma membrane integrity (SPMI) ≥ 70%, and sperm acrosome integrity (SAI) ≥ 80%.

### Sperm diluent

A tris-citric-acid-fructose (TCF) solution was created using the ingredients, following our previous study [14]: tris 3.41 gm (Himedia Lab. Pvt., Ltd.; Mumbai 400086; India), citric acid 1.61 gm (Sigma-Aldrich Corp.; St. Louis, MO 63103; USA), and fructose 0.81 gm (Himedia Lab. Pvt., Ltd.) with deionized water (RSL Lab. Ltd., Bangkok 10330, Thailand). The subsequent extenders were made independently, without or in addition to D (+)-glucose (G) and trehalose (T) to the TCF solution: control (neither G nor T), G-75, G-150, T-75, and T-150 mm. Following thorough mixing at 50°C–70°C and 30–200 rpm for 15 min, the extenders were cooled to 4°C before the addition of gentamicin (250 µg/ml) and 16% (v/v) egg yolk. Each extender was adjusted to a final volume of 100 ml by adding deionized water while maintaining a pH of 7.00. The extenders were then mixed using a magnetic stirrer, gradually increasing the speed from 30 to 200 rpm without heating. Afterward, they were centrifuged at 3,000 rpm for 15 min at room temperature, and the supernatant was carefully collected. Each group of extenders was further used for cool semen storage.

### Sperm progression and total motility (TM)

Sperm progressive motility and sperm total motility were assessed after mixing 10 µl of sperm with prewarmed (37°C) washing media composed of tris (3.41 gm), citric acid (1.61 gm), fructose (0.81 gm), gentamicin (250 µg/ml), and deionized water up to 100 ml (pH 7.0). A prewarmed coverslip was placed over a 10 µl sperm sample on a clean, prewarmed glass slide. A total of 200 sperm cells were analyzed under a bright-field microscope at 400× magnification across at least three different fields. Progressive motility (PM) and non-PM were recorded following the World Health Organization 2010 guidelines.

### Sperm viability

Based on prior research [14,15], semen vitality was assessed using the eosin-nigrosin staining method. The staining solution was prepared by dissolving 1% eosin (Sigma-Aldrich Corp.), 5% nigrosin (Himedia Lab. Pvt., Ltd.), and 3% trisodium citrate dihydrate (Himedia Lab. Pvt., Ltd.) in distilled water. The solution was prepared with gentle heating and continuous stirring, ensuring no boiling occurred. Once fully dissolved, it was cooled and stored at room temperature in a 15-ml Falcon tube wrapped in aluminum foil. A 10-µl semen sample was thoroughly mixed with 10 µl of the staining solution in an Eppendorf tube and left undisturbed for at least 30 sec. Following this, 10 µl of the stained sample was placed on a slide, covered with a coverslip, and allowed to settle before evaluation. Using a bright-field microscope at 400× magnification, three separate fields were examined, analyzing a total of 200 sperm cells. Live sperm were identified by unstained (white) heads, while dead sperm exhibited pink or red-stained heads.

### Normal sperm (NS)

The normal morphology of sperm was identified using the eosin-nigrosin staining technique, from our earlier research [14,15]. In accordance with Ayesha *et al*. [16], the normal and abnormal sperm were described. Sperm with narrow, small, double heads, broken, short mid-pieces, coiled, bent, and short tails were considered abnormal sperm of bucks. A bright-field microscope (400×) was used to analyze 200 sperm across at least three distinct fields, and the percentage of NS was recorded.

### Sperm plasma membrane integrity

Semen functional integrity (FI) was determined using the hypoosmotic swelling test (HOST) following the staining procedure described in prior studies [14,15]. In this method, 62.5 µl of semen was incubated with 250 µl of HOST solution at 37°C for 1 h. After incubation, a 10 µl semen sample was combined with 10 µl of staining solution containing 1% eosin, 5% nigrosin, and 3% trisodium citrate dihydrate in distilled water. The stained sample was placed on a prewarmed slide and left undisturbed for 30 sec before being covered with a coverslip preheated to 37°C. The proportion of sperm displaying tail swelling or coiling was assessed under a light microscope at 400×, with at least 200 sperm examined across three distinct fields.

### Sperm acrosomal integrity

SAI was assessed using the method described by Kamal *et al*. [14]. A 1% formal citrate solution was prepared by dissolving 1% (v/v) commercial formaldehyde (37%) (Merck, Germany) and 2.9% (w/v) trisodium citrate dihydrate (Himedia Lab. Pvt., Ltd.) in distilled water. A 250 µl semen sample was fixed by adding 25 µl of the prepared formal citrate solution. Subsequently, 10 µl of the fixed sperm suspension was transferred onto a slide, evenly smeared, and covered with a coverslip for further examination. 100 sperms were inspected under a 1,000× light microscope to see the typical apical ridge. Sperms were normal if their acrosome area was normal, smooth, and crescentic.

### Experimental design

This study evaluated the impact of varying glucose and trehalose concentrations on the preservation of high-quality buck sperm during cooled storage. The collected ejaculate was carefully pipetted into five separates, clearly marked Falcon tubes and divided into five equal groups. Control (0%, G, and T), G-75, G-150, T-75, and T-150 were written on the tubes. Each group’s final concentration was set at 9 × 10^7^ spermatozoa/ml during separation. Each identified sperm tube was stored in a refrigerator at 4°C. After dilution, sperm quality was evaluated at 5, 24, 48, and 72 h of cooled storage to assess the effects of different glucose and trehalose concentrations. At each storage interval, a 40 µl semen aliquot was carefully transferred into prewarmed tris-based washing media and incubated at 37°C in a water bath for 5 min. The percentages of PM, TM, SV, NS, SPMI, and SAI were analyzed across all five extender groups.

### Statistical analysis

The total observation of the experiments = each sperm parameter observation × sperm parameter numbers × sperm observation periods = 6 × 6 × 5 = 150 times, and the findings were displayed as means ± SE. Statistical differences in semen parameters were analyzed using ANOVA followed by the Tukey-HSD test (SPSS version 26.0, SPSS Inc., Chicago, IL). A significance level of *p *< 0.05 was applied in all cases.

## Results

**Figure 1.**
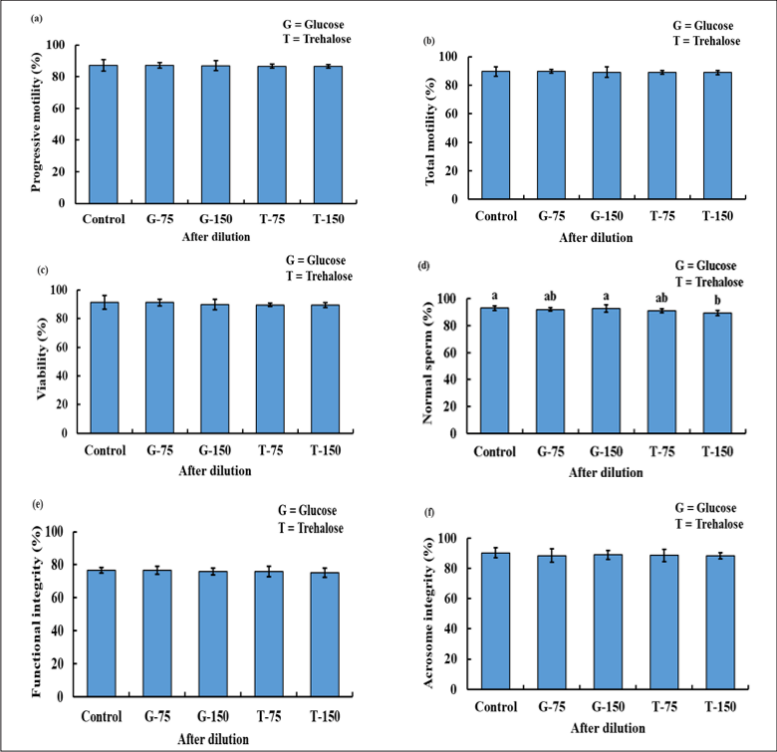
The PM (a), TM (b), viability (c), NS (d), FI/plasma membrane integrity (e), and acrosome integrity (f) status of bucks after being used with or without various concentration of glucose and trehalose in TCEF extender after dilution.

Following dilution, no significant differences were observed in sperm PM, TM, SV, SPI/FI, and acrosome integrity among the control and the G-75, G-150, T-75, and T-150 groups. However, the NS of T-150 was significantly lower compared to the control and G-150 (Fig. 1).

**Figure 2.**
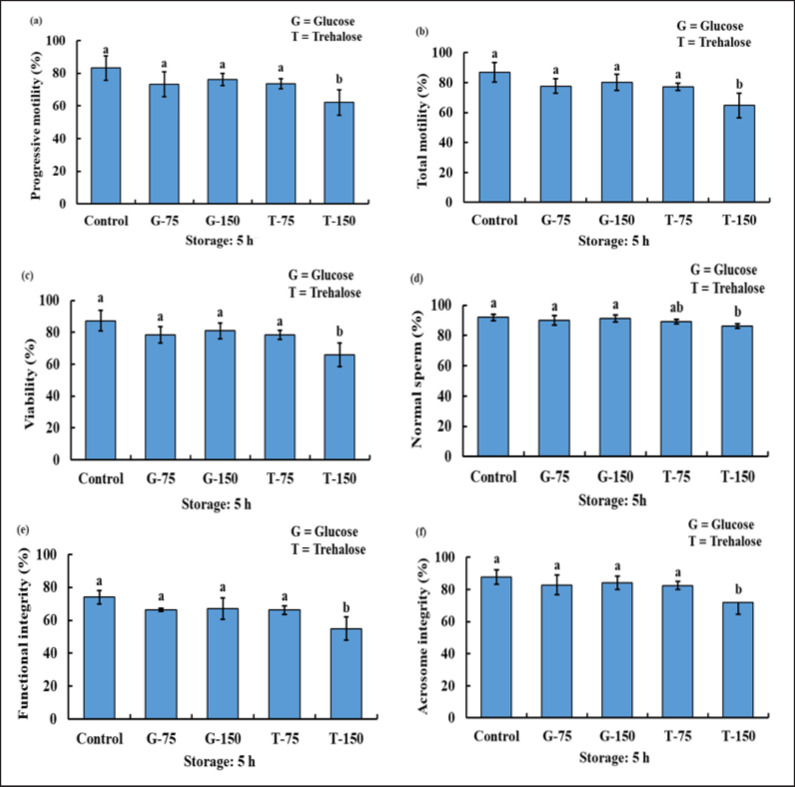
The PM (a), TM (b), viability (c), NS (d), FI/plasma membrane integrity (e), and acrosome integrity (f) status of bucks after being used with or without various concentration of glucose and trehalose in TCEF extender after 5 h of storage.

**Figure 3.**
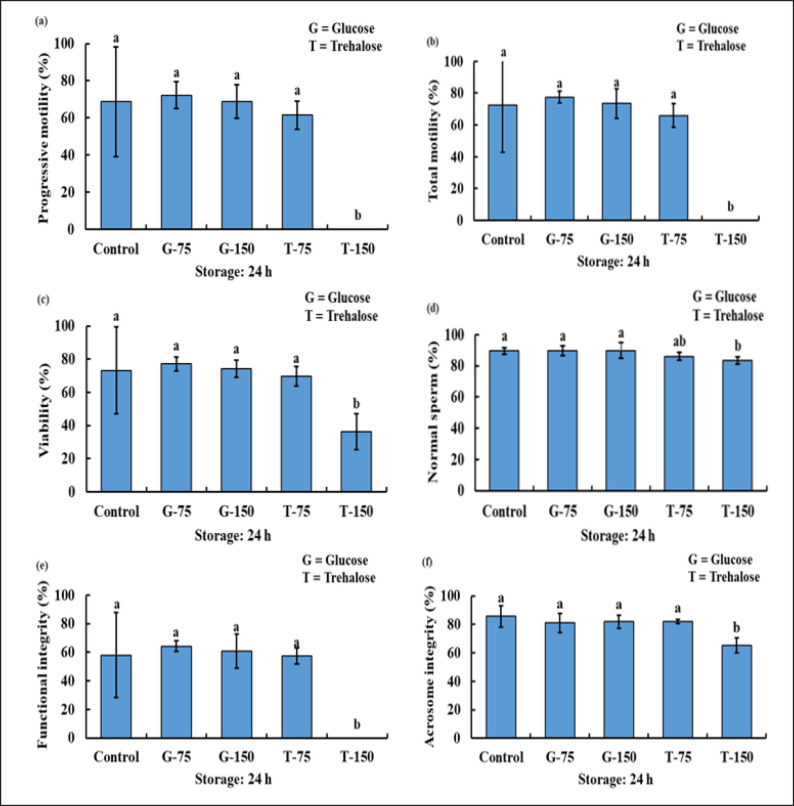
The PM (a), TM (b), viability (c), NS (d), FI/plasma membrane integrity (e), and acrosome integrity (f) status of bucks after being used with or without various concentration of glucose and trehalose in TCEF extender after 24 h of storage.

After 5 and 24 h of cool storage, the PM, TM, SV, SMI/FI, and acrosome integrity of the sperm of T-150 were significantly decreased compared to the control, G-75, G-150, and T-75. Nevertheless, there are no notable variations in the PM, TM, SPMI/FI, and acrosome integrity of the sperm among the control, G-75, G-150, and T-75. Whereas the NS of T-150 was considerably lower than that of the control, G-75, and G-150. In addition, the NS for the control, G-75, G-150, and T-75 did not differ significantly (Figs. 2 and 3).

**Figure 4.**
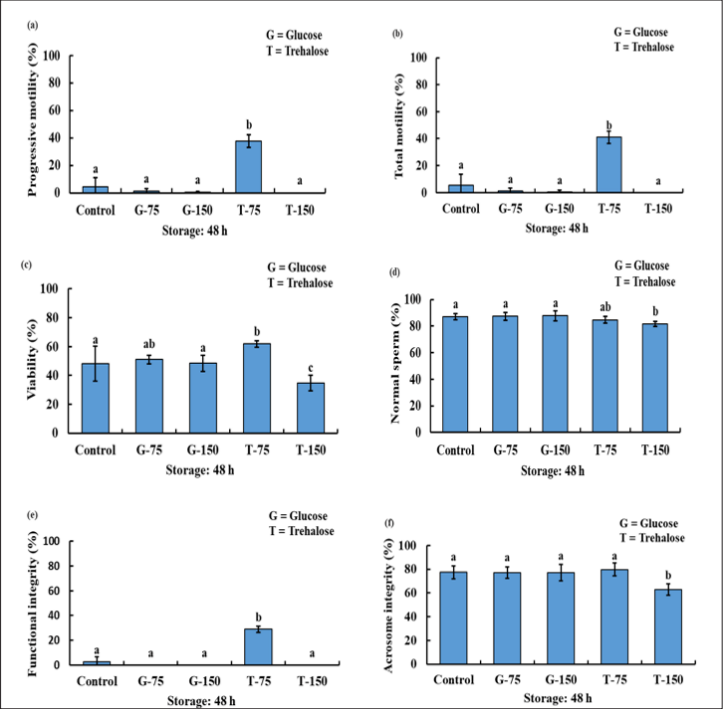
The PM (a), TM (b), viability (c), NS (d), FI/plasma membrane integrity (e), and acrosome integrity (f) status of bucks after being used with or without various concentration of glucose and trehalose in TCEF extender after 48 h of storage.

After 48 h of cool storage, the T-75 provided the best result, and the sperm PM, TM, and SPMI/FI of T-75 were significantly increased compared to the other groups. The SV of T-75 significantly increases compared to the control, G-150, and T-150. However, there were no notable variations in SV between G-75 and T-75. In contrast, the NS of T-75 was not much different from all the other groups. However, the acrosome integrity of T-150 was substantially less than the control, G-75, G-150, and T-75 (Fig. 4).

**Figure 5.**
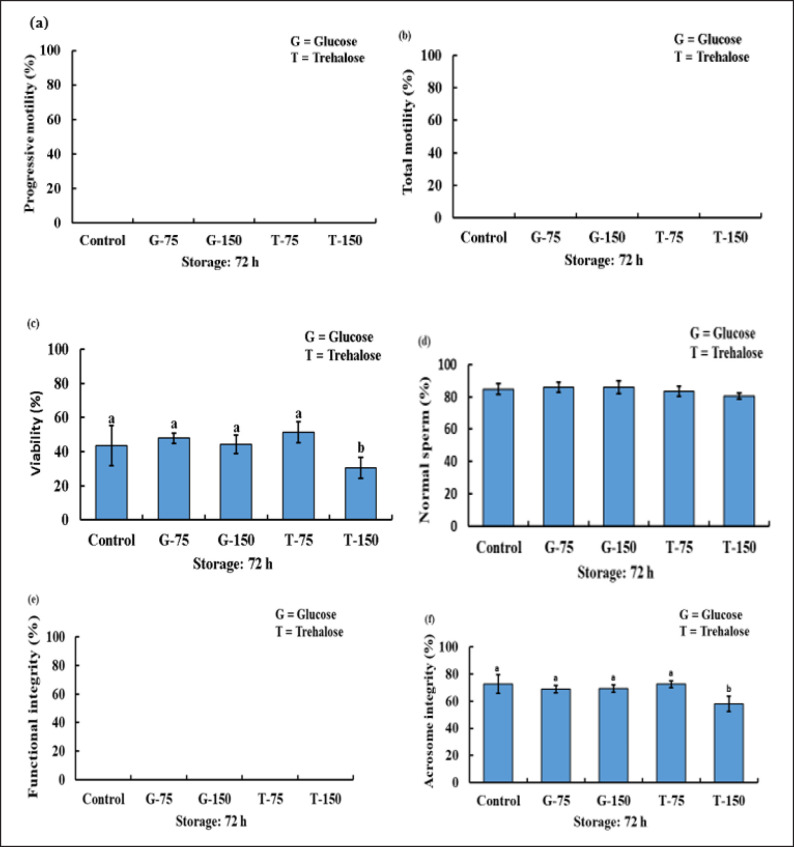
The PM (a), TM (b), viability (c), NS (d), FI/ plasma membrane integrity (e), and acrosome integrity (f) status of bucks after being used with or without various concentration of glucose and trehalose in TCEF extender after 72 h of storage.

After 72 h of cool storage, there were no PM, TM, and SPMI/FI of sperm in all the study groups. The viability of semen of T-150 was considerably lower than the control, G-75, G-150, and T-75. In the case of NS, there were no notable differences among the groups. In comparison to the control, G-75, G-150, and T-75 sperm, the acrosome integrity of the T-150 sperm was considerably reduced (Fig. 5).

## Discussion

The main goal of adding exogenous sugar to a sperm extender is to supply energy for sperm metabolism and function. For effective fertilization, SM, viability, morphology, PMI, and acrosome integrity are essential. Animal semen stored in a cool environment undergoes structural and functional alterations, resembling the natural aging process, which varies based on species, storage temperature, and duration [17]. In this work, the effectiveness of goat sperm after dilution and cool storage was examined at different ratios of glucose and trehalose in TCEF diluents. This investigation’s findings reveal notable insights into the impact of these additives on various sperm parameters over different durations of cold storage.

According to the study’s findings, adding trehalose to the TCEF extender at a 75 mm concentration enhanced motility, viability, and FI of the preserved semen up to 48 h of cool storage. In a previous study, trehalose supplements considerably enhanced the semen quality of the Duolang ram semen. Whereas trehalose supplementation significantly improved the level of viability and PMI in comparison with the control group at 0°C [3]. Furthermore, trehalose can serve as a protective agent against oxidative damage, desiccation, and high temperatures. It can create a barrier that protects cells and biomolecules. According to previous studies, it possesses anti-inflammatory and antioxidant qualities [12] that improve sperm quality.

The current research has shown that increasing trehalose concentration to 150 mm (T-150) did not enhance semen parameters. A recent study found that ram spermatozoa can withstand a hyperosmotic extender at 50–100 mm levels of sugar, improving the quality of post-thaw semen [18]. SM is affected by external osmolarity [19]; high sugar concentrations provide the extender’s high osmolality, which damages the semen cells [13]. This is in agreement with a recent investigation’s findings, which showed that ram SM and FI after thawing were negatively impacted by trehalose at 200 and 400 mOsm [19]. Moreover, there was a noticeable decrease in the semen functional membrane in rams when employing hypertonic diluents (tris-citrate modified solution) and 76 gm/l trehalose [20]. Interestingly, our previous study found that the semen cryopreservation in bucks, the T-150, provides the best outcome compared to low or high supplements of trehalose in the TCEF diluents with 7% glycerol. A previous study also found that adding 375 mm of 4% glycerol with trehalose to the diluents improved the quality of the sperm after Shiba bucks were frozen [21]. This is due to the cryopreservation used at a lower temperature than the cool storage of sperm at 4°C [5,22].

During cryopreservation, water inside the cells can form ice crystals, leading to dehydration of the cells and potential damage [23,24], thus, trehalose binds to the sperm cell membrane following treatment, reducing sperm damage from dehydration and damage to the plasma membranes and stabilizing the composition of the membrane of semen cells [12]. On the other hand, the cool storage slows down the metabolic processes and reduces bacterial growth, rather than freezing the sperm [25]. So, it is indicated that the concentration of trehalose or other cryoprotectants needed during cryopreservation of sperm is higher compared to cool storage at 4°C due to the more extreme conditions involved in freezing and the need for greater protection against cellular damage. Whereas the greater trehalose concentrations increased the viscosity of the semen, which lowered its quality of semen after cool storage. Thus, following cool storage, sperm properties can be improved by adding the appropriate amount of trehalose to the fresh buck semen. So, our findings show that the addition of trehalose (75 mm) to the TCEF diluent enhances the quality of fresh buck semen after 48 h of cooled storage at 4°C.

Conversely, glucose supplementation at 75 and 150 mm showed relatively consistent performance for sperm quality throughout the 24 h period, with no significant differences observed compared to the control and T-75 groups. However, the PM, TM, SV, FI, and acrosome integrity were decreased in the T-150 compared to the G-75, G-150, and T-75. This data suggests that glucose may provide a stable environment for sperm preservation up to 24 h cold storage, possibly due to its role as a primary energy source for sperm metabolism [9]. However, after 48 h of cold storage, the PM, TM, and FI were considerably reduced in the G-75 and G-150 compared to the T-75. When glycerol was absent from the diluents, frozen-thawed sperm showed greater motility with trehalose compared to glucose; however, no significant differences were observed when glycerol was present [14].

As per a study [26], disaccharides such as sucrose and trehalose in boar have been found to be better cryoprotective than monosaccharides like glucose, galactose, or fructose. On the other hand, a distinct investigation revealed that in comparison to trehalose or sucrose supplementation, glucose supplementation greatly enhanced (*p* < 0.05) the forward movement of buck semen following freezing [27]. The SM, PMI, and acrosome integrity were all successfully preserved during cooling storage in the stallion semen by raising the glucose content in the extender to 40 mm [28]. In this context, supplementing the tris-egg yolk extender with 70 mm glucose and 70 mm fructose significantly improved chilled canine sperm quality compared to 10 mm, resulting in higher motility and VAP values throughout the experiment [4]. On the other hand, ram sperm can remain viable for fertilization after being cooled and preserved at 0°C when treated with 5 mm trehalose. However, trehalose concentrations below 20 mm in the tris-fructose egg yolk medium had no significant effect on improving PM, TM, or SAI [3].

Nevertheless, the earlier research indicated that the monosaccharides and disaccharides were found to improve the SM from bucks and rams during the first 3 h of *in vitro* incubation in tris medium. Diverse spermatozoa, sperm media, and sugar supplements used for enhancement can lead to varied patterns of motility [29]. Therefore, the effectiveness of glucose and trehalose in sperm freezing seems to depend on factors such as species, temperature, and potentially the specific techniques used, and T-75 was more suitable than G-75 and G-150 due to the beneficial effect of T-75 described above.

Overall, our findings recommended that the T-75 mm employed in the TCEF-containing extender can significantly improve the efficiency of goat sperm cool storage up to 48 h of storage. However, glucose 75 and 150 mm have a potential effect up to 24 h of cool storage of buck sperm. Further studies are warranted to elucidate the underlying mechanisms at the molecular level and optimize the use of additives for semen preservation in goats and potentially other species.

## Conclusion

Trehalose, particularly at a concentration of 75 mm, was more effective than glucose in maintaining sperm quality during short-term cold storage of buck sperm. The T-75 group exhibited superior PM, TM, and PMI up to 48 h. After 72 h, sperm quality declined across all groups, with T-150 showing the most detrimental effects. Therefore, we recommend trehalose at 75 mm to preserve goat semen optimally for up to 48 h.

## List of abbreviations

AI, Artificial insemination; °C, degree Celsius; FI, Functional integrity; G, Glucose; gm, gram; h, hour; HOST, Hypoosmotic swelling test; IAMEBBC, Institutional Animal, Medical Ethics, Biosafety, and Biosecurity Committee; kg, kilogram; ml, milliliter; mm, millimole; NS, Normal sperm; PM, progressive motility; RPM, revolutions per minute; SAI, Sperm acrosome integrity; SPMI, Sperm plasma membrane integrity; STM, Sperm total motility; SV, Sperm viability; T, Trehalose; TCEF, Tris-citric acid-egg-yolk-fructose; TCF, tris-citric-acid-fructose; TM, total motility; %, percentage; µl, microliter;µg, microgram.
